# 肿瘤细胞与神经信号在神经内分泌癌转移中的串扰作用：基于通讯劫持的视角

**DOI:** 10.3779/j.issn.1009-3419.2025.101.03

**Published:** 2025-03-21

**Authors:** Shuping SONG, Xinyi WANG, Siqi ZHOU, Xuchen CHENG, Weixuan LIN, Yongxuan WANG, Yanqin SUN

**Affiliations:** ^1^523808 东莞，广东医科大学附属东莞松山湖中心医院肿瘤中心; ^1^Department of Cancer Center, The Affiliated Dongguan Songshan Lake Central Hospital of Guangdong Medical University,Dongguan 523808, China; ^2^524000 湛江，广东医科大学病理学系; ^2^Department of Pathology, Guangdong Medical University, Zhanjiang 524000, China; ^3^524000 湛江，广东医科大学第一临床医学院; ^3^The First Clinical Medical College, Guangdong Medical University, Zhanjiang 524000, China

**Keywords:** 癌症神经科学, 肿瘤转移, 神经内分泌癌, 串扰作用, 通讯劫持, Cancer neuroscience, Tumor metastasis, Neuroendocrine carcinoma, Crosstalk effect, Communication hijacking

## Abstract

神经内分泌癌（neuroendocrine carcinoma, NEC）为一类起源于神经内分泌细胞的恶性肿瘤。由于NEC细胞兼具神经细胞和内分泌细胞的特性，在肿瘤转移过程中可通过多种机制劫持神经元信号传导并动态调节神经元谱系标志物的表达，从而形成有利于肿瘤生长与转移的微环境。另一方面，肿瘤微环境的变化反过来也会增强神经元与肿瘤细胞的相互作用，最终协同促进NEC转移的发生。本综述介绍了癌症神经科学领域的研究进展，揭示了NEC中的神经元谱系标志物，通过介导肿瘤细胞与神经系统之间发生串扰、双向交流以及协同作用，促进肿瘤扩散转移。因此，肿瘤神经科学领域的最新研究成果，丰富了我们对肿瘤转移生物学机制的认识，为深入理解肿瘤转移，特别是脑转移的复杂生物学过程，开辟了新的研究方向。本文就肿瘤细胞与神经信号在NEC转移中的串扰作用作一综述。

## Abstract (en)

Neuroendocrine carcinoma (NEC) represents a category of malignant tumors originating from neuroendocrine cells. Given that NEC cells exhibit characteristics of both neural and endocrine cells, they can hijack neuronal signaling pathways and dynamically regulate the expression of neuronal lineage markers during tumor metastasis, thereby constructing a microenvironment conducive to tumor growth and metastasis. Conversely, alterations in the tumor microenvironment can enhance the interactions between neurons and tumor cells, ultimately synergistically promoting the metastasis of NEC. This review highlights recent advancements in the field of cancer neuroscience, uncovering neuronal lineage markers in NEC that facilitate tumor dissemination through mediating crosstalk, bidirectional communication, and synergistic interactions between tumor cells and the nervous system. Consequently, the latest findings in tumor neuroscience have enriched our understanding of the biological mechanisms underlying tumor metastasis, opening new research avenues for a deeper comprehension of the complex biological processes involved in tumor metastasis, particularly brain metastasis. This review provides a comprehensive review of the crosstalk between tumor cells and neural signaling in the metastasis of NEC.

肿瘤转移是指恶性肿瘤细胞从原发灶侵入淋巴管、血管或体腔，向其他部位迁移并继续生长，形成继发性肿瘤，其性质与原发肿瘤相同^[1,2]^。恶性肿瘤的转移在其恶性生物学行为中发挥的作用巨大，这一过程不仅是影响癌症患者预后的重要因素，也是造成癌症相关死亡的主要原因之一^[3]^。数据^[4]^显示，大约90%的癌症患者因转移性疾病而死亡，其中脑转移是尤为严重且复杂的转移形式之一。随着多种癌症发病率的上升，脑转移的发生率也在逐渐增加。脑转移是最常见的神经系统并发症之一，发病率为9%-17%^[5]^，其临床症状包括头痛、癫痫发作、认知功能障碍以及神经功能缺失等，这些症状严重影响患者的生活质量和生存期。

神经内分泌癌（neuroendocrine carcinoma, NEC）在不同癌症类型中表现出显著的异质性^[6,7]^，NEC的细胞不仅具备分泌激素的能力，还展现出显著的转移潜能，特别是对脑组织的亲和性逐渐成为研究焦点^[8]^。NEC细胞通过多种机制促进脑转移的发生，这些机制包括细胞间相互作用、微环境的改变以及基因表达调控等。

在当前研究背景下，本综述旨在探讨NEC的生物学特性及其转移的分子机制，期望能够揭示NEC脑转移的潜在生物标志物及治疗靶点，为后续研究提供科学依据。

## 1 癌症转移的基本机制

### 1.1 癌症转移的常见途径及其特点

癌症转移常见的转移形式包括血行转移、淋巴道转移和直接浸润^[3]^。血行转移是转移方式中最为普遍的途径，恶性肿瘤侵入血管后可随血液到达远处器官继续生长，最终在远处器官如肺、肝、骨骼等形成转移性病灶。淋巴道转移是上皮性恶性肿瘤转移的最常见方式。恶性肿瘤内部一般没有淋巴管，淋巴道转移通常通过肿瘤旁的引流淋巴管，瘤细胞侵入淋巴管后，随淋巴液回流首先到达局部引流淋巴结，然后聚集于边缘窦，继续增殖继而累及整个淋巴结，有时多个淋巴结融合成肿块，接着肿瘤可转移到下一站淋巴结。最终肿瘤可经胸导管进入血流，从而出现血行转移^[9]^。直接浸润则是肿瘤细胞通过周围组织的扩展，直接侵犯邻近器官或组织。这些转移途径的特征在于肿瘤细胞在不同的微环境中展现出不同的适应性机制。例如，在骨髓微环境中，肿瘤细胞可能经历一种“肿瘤休眠”状态^[10]^，以逃避免疫系统的监视并形成潜在的转移性病灶^[3]^。此外，肿瘤细胞在转移过程中能够通过改变细胞外基质的组成和功能，促进自身的存活和增殖，从而加速转移过程的发生。

### 1.2 肿瘤微环境（tumor microenvironment, TME）因素在癌症远处转移中的作用

在癌症转移过程中，TME发挥着关键性作用^[11]^。TME是由肿瘤细胞及其周围的成纤维细胞、免疫细胞/炎症细胞、细胞因子、血管内皮/血管周细胞、细胞外基质等相互作用形成的、有利于肿瘤生存及演进的特殊局部环境。该TME内的细胞组分，包括肿瘤相关成纤维细胞、免疫细胞以及内皮细胞，通过分泌细胞因子、化学介质和生长因子，对肿瘤细胞的行为产生显著影响^[12]^。有研究^[13]^显示，肿瘤相关的免疫细胞/炎症细胞及其介质可能直接参与肿瘤的发生发展，对肿瘤免疫产生重要影响，形成十分重要的“肿瘤免疫微环境”（tumor immune microenvironment, TIME）。此外，TME中的缺氧状态亦被视为促进肿瘤细胞转移的关键因素之一^[14]^，低氧条件不但引起代谢变化，而且影响多个信号途径关键分子的表达，促进肿瘤的生长^[15]^。低氧亦可诱导肿瘤细胞经历上皮-间充质转化（epithelial-mesenchymal transition, EMT），进而令肿瘤细胞具备更为强劲的侵袭转移能力^[16,17]^。TME的物理特性^[18]^，例如细胞外基质改建，同样对肿瘤细胞的迁移与转移能力产生影响^[19,20]^。因此，深入理解TME的复杂性及其对转移过程的作用，对于研发新的治疗策略具有至关重要的意义^[12]^。

### 1.3 脑内微环境在肿瘤脑转移时的适应机制

脑内微环境的特异性对肿瘤细胞转移过程具有重要影响^[21]^。脑组织的结构与功能特性，例如血脑屏障的存在和神经细胞的高密度分布，共同构建了一个相对隔绝且复杂的微环境，为肿瘤细胞侵入脑组织带来了多重挑战^[22]^。脑内微环境的特征主要体现在神经元和胶质细胞的丰富性、细胞外基质成分的特定性以及血管结构的独特性，这些因素为肿瘤细胞的生长和扩散提供了特定的环境条件^[23]^。研究^[24]^表明，在转移过程中，肿瘤细胞通过调整其表面标志物表达和分泌特定细胞因子来适应微环境，从而促进其存活和增殖。此外，中枢神经系统微环境的动态变化亦可能对肿瘤细胞的侵袭性和转移潜能产生影响，例如，特定细胞因子表达的上调可能促进肿瘤细胞的迁移和侵袭^[25]^。因此，深入理解中枢神经系统微环境的特性以及肿瘤细胞在转移过程中的适应机制，对于开发新的治疗策略具有至关重要的意义。

研究^[26⇓-28]^发现，NEC细胞在转移到脑组织后，能够与脑内微环境中的神经元和胶质细胞进行相互作用，从而改变其生物行为和转移特性。此类肿瘤细胞通常展现出较高的侵袭性以及对脑微环境的适应性，例如，通过分泌神经递质或细胞因子以促进自身的生长和存活^[29]^。此外，NEC的转移与脑内免疫微环境紧密相关，肿瘤细胞可能通过调节免疫细胞的功能，逃避宿主的免疫监视，进而促进转移过程^[30,31]^。因此，深入探讨NEC在脑转移过程中的微环境特征对于深入理解其转移机制具有重要意义，并且对于制定有效的治疗策略至关重要。接下来，我们将对两种常见的NEC，即小细胞肺癌（small cell lung cancer, SCLC）和前列腺癌的脑转移机制进行具体阐述。

SCLC是一种恶性程度极高、预后很差的肺癌类型，其转移至脑部的机制复杂^[32⇓-34]^。SCLC细胞能够诱导反应性星形胶质细胞募集到TME中。SCLC细胞与星形胶质细胞之间的相互作用促进了基因表达程序的激活，这些程序与大脑发育早期神经元与星形胶质细胞相互作用时所发现的基因表达模式相似^[35]^。在分子机制层面，SCLC细胞分泌的大脑发育相关因子，将星形胶质细胞募集到脑转移瘤中。这些星形胶质细胞又反过来释放促存活因子进一步促进SCLC细胞的增殖^[36]^。因此，SCLC脑转移瘤的生长机制涉及了大脑发育过程中神经元与星形胶质细胞相互作用的机制^[37,38]^。另有研究^[39⇓⇓-42]^表明，SCLC细胞通过多种途径穿越血脑屏障，导致脑转移的发生，提示SCLC细胞在脑转移过程中与脑微环境的相互作用，强调了针对这些机制的潜在治疗策略的重要性。

前列腺癌是男性泌尿生殖系统最常见的恶性肿瘤，其转移途径主要包括淋巴转移和血行转移。前列腺癌细胞能够通过淋巴系统向盆腔和腹股沟淋巴结转移，随后可能进一步转移至骨骼等远处器官。对于目前研究，雄激素剥夺治疗（androgen deprivation therapy, ADT）仍是前列腺癌的主要治疗手段^[43]^，但近年来的研究表明，由于神经内分泌前列腺癌（neuroendocrine prostate cancer, NEPC）细胞谱系可塑性^[44]^，使得前列腺腺癌向神经内分泌前列腺癌转分化，并极易出现ADT抵抗甚至耐药^[45,46]^。大部分患者在内分泌治疗12-18个月后，进展为更具侵袭性的去势抵抗性前列腺癌（castration resistant prostate cancer, CRPC），并有神经内分泌化的形态出现，由腺癌向神经内分泌前列腺癌转化^[47]^。有研究^[48,49]^发现，23例前列腺腺癌新发或转化发生的大细胞神经内分泌前列腺癌病例中有6例出现脑转移，表明NEC和脑转移具有相关性。此外，在前列腺癌中神经肽Y及其受体表达与癌症的侵袭性及神经周围转移密切相关，提示在前列腺癌转移中这些分子所起到的潜在作用^[50]^。这些发现提供了一个新的视角来理解前列腺癌的转移机制，并提供了一个理论基础来发展靶向疗法。

## 2 通讯劫持在肿瘤转移中的作用

在肿瘤转移过程中，通讯劫持发挥着至关重要的作用，特别是在肿瘤细胞与邻近神经元的相互作用中^[51]^。肿瘤通讯劫持是指肿瘤细胞通过各种机制劫持正常细胞的信号通路，从而促进自身的生长、存活和转移^[52]^。研究^[53]^表明，肿瘤细胞能够通过多种机制劫持神经元的信号传导途径，进而促进其自身的增殖与转移。肿瘤细胞通过分泌神经递质及其他信号分子，能够干预神经元的功能，改变神经元的电生理特性，从而为肿瘤细胞的存活与扩散营造有利的TME^[26]^，如胶质母细胞瘤，可利用神经元的信号传递机制提升其侵袭性和转移潜能。

SCLC细胞同时具备激素分泌细胞和神经元的特性，这为其增殖和扩散提供了优势^[40]^。SCLC细胞通过劫持自分泌的KIT、Hedgehog和IGF1信号通路，增强自身增殖和转移能力^[54]^。此外，神经内分泌型与非神经内分泌型SCLC细胞之间的旁分泌FGF信号可能促进了细胞的存活和转移^[55]^。部分SCLC患者表现出的内分泌副瘤综合征，暗示了SCLC细胞与体内其他细胞之间存在远程通信机制^[56]^。

前列腺癌细胞通过分泌白细胞介素6（interleukin 6, IL-6）和IL-8等多种细胞因子劫持免疫微环境，从而促进肿瘤的进展和免疫逃逸^[57]^。IL-6通过激活p38丝裂原活化蛋白激酶（p38-mitogen-activated protein kinase, p38-MAPK）信号通路，驱动前列腺癌的神经内分泌分化，这种分化与肿瘤的侵袭性和耐药性密切相关^[58]^。

此外，神经元与肿瘤细胞之间的相互作用亦涉及微胶质细胞的调控，这些细胞在TME中扮演着关键角色，能够影响肿瘤的生长和转移。

### 2.1 神经元与肿瘤细胞的相互作用

神经元与肿瘤细胞之间的相互作用在肿瘤转移过程中扮演着至关重要的角色^[26,53]^。肿瘤细胞通过劫持神经元的信号传导途径，能够获取其生长所需的营养物质及支持。研究^[59]^发现，肿瘤细胞通过分泌神经递质，激活神经元内的信号传导途径，进而促进其自身的增殖与迁移。例如，胶质母细胞瘤细胞能够通过与神经元的直接相互作用，改变神经元的电生理特性，促使神经元释放更多的神经递质，从而进一步促进肿瘤的生长^[60]^。此外，神经元的信号传递过程亦可能影响肿瘤细胞的基因表达模式，导致肿瘤细胞展现出更强的转移潜能^[61]^。这些相互作用不仅为肿瘤细胞提供了生长信号，还可能改变局部微环境的构成，从而促进肿瘤的进一步扩散。

### 2.2 肿瘤相关信号通路的变化与脑转移相互促进

肿瘤相关信号通路的变化与脑转移之间存在着复杂的相互促进关系。在转移过程中，肿瘤细胞激活了包括PI3K/Akt、MAPK/Erk在内的多种信号通路，这些通路不仅促进了肿瘤细胞的增殖与存活，还可能对神经元的功能与行为产生影响^[62]^。例如，研究^[63]^揭示，肺癌细胞通过分泌外泌体中的miRNA，能够调节神经元的信号通路，进而增强其转移能力。此外，非小细胞肺癌细胞代谢状态的改变亦会影响其与神经元的相互作用，从而促进肿瘤细胞穿越血脑屏障，形成脑转移，与脑转移相关的肺癌驱动基因对患者的预后具有显著影响^[64,65]^。通过深入研究这些驱动基因及其信号通路，可以更准确地识别易发生脑转移的肺癌患者。因此，肿瘤细胞与神经元之间的相互作用以及肿瘤相关信号通路的改变，共同构成了一个复杂的网络，促进了肿瘤的转移与扩散。

## 3 神经信号与肿瘤细胞在脑转移中的串扰作用

### 3.1 神经信号异常与肿瘤发生发展

在肿瘤转移过程中，神经信号的异常通常表现为神经元谱系标志物表达的改变，此现象在脑转移性肿瘤中尤为突出^[66]^。研究^[67]^发现，特定神经元谱系标志物如嗜铬粒蛋白A（chromogranin A, CHGA）、突触素（synaptophysin, SYP）和CD56的表达增加，可能与肿瘤细胞的恶性程度及转移潜能相关联。ZEB1作为转录抑制因子，能够抑制神经分化，并与CTBP2协同动态调节细胞迁移，这对于解析大脑皮质发育的精细调控机制至关重要^[68]^。此外，miR-124的肿瘤抑制作用与其在神经发育中的功能紧密相关，提示神经谱系标志物的异常表达可能对肿瘤细胞的生物学特性产生影响^[69]^。同时，微环境的改变亦可导致肿瘤细胞对神经谱系标志物表达的调控，进而影响肿瘤的进展和转移能力^[70]^。因此，深入探讨神经元谱系标志物在肿瘤发生发展中的异常表达机制，对于揭示脑转移的生物学特性具有重要的科学意义。

### 3.2 神经元谱系标志与肿瘤细胞的串扰作用对肿瘤转移的影响

肿瘤细胞的串扰作用是指肿瘤细胞与TME中的其他细胞（如免疫细胞、成纤维细胞等）之间的复杂相互作用。这些相互作用通过多种信号通路和细胞因子进行，显著影响肿瘤的发生、进展、转移和免疫抑制^[71]^。神经元谱系标志与肿瘤细胞之间的串扰作用在肿瘤转移过程中发挥着重要的调控作用^[72]^。研究^[70]^表明，TME中的神经元谱系标志能够通过多种机制促进肿瘤细胞的转移。例如，胶质细胞分泌的可溶性因子已被发现可以增强胰腺腺癌细胞的转移潜能，并诱导EMT。这种转化使得肿瘤细胞获得更强的侵袭性和迁移能力，从而促进脑转移的发生。此外，肿瘤细胞与神经元谱系细胞之间的相互作用，也可能通过改变细胞信号通路，影响肿瘤的生长和转移特性。例如，交感神经元可以和SCLC细胞发生串扰作用，交感神经元通过β2肾上腺素能受体促进SCLC的生长^[73]^。肿瘤细胞通过调控神经元谱系标志的表达，可能会改变其微环境，从而促进自身的生存和扩散^[74]^。因此，理解神经元谱系标志与肿瘤细胞之间的串扰机制，对于开发新的治疗策略以抑制脑转移具有重要的临床意义。

在近年来的癌症神经科学研究中，科学家们取得了一系列关键性发现，逐步揭示了肿瘤与神经系统之间错综复杂的相互作用机制。神经内分泌转化作为一种谱系可塑性的表现形式，与表观遗传重编程紧密相关，并且可能在强效靶向治疗的暴露下得到促进或被选择性增强^[75]^。尽管已明确TP53和RB1两种肿瘤抑制基因的共同突变失活是神经内分泌转化的前提条件，但关于驱动该转化的分子机制的了解仍然有限^[76,77]^。即便能够识别出具有高风险发生神经内分泌转化的腺癌患者，目前仍缺乏有效的策略来抑制谱系可塑性和预防神经内分泌转化。特别是在脑转移的背景下，通讯劫持与串扰作用已被证实为肿瘤细胞在中枢神经系统内生长和扩散的关键机制^[78]^。这些研究发现不仅深化了我们对癌症生物学的认识，也为临床治疗提供了新的视角。在该研究领域，研究者们特别关注肿瘤细胞通过劫持神经元间的信号通信来促进自身的存活和增殖。例如，肿瘤细胞能够释放特定的细胞因子，干扰神经元的正常功能，进而改变微环境，为肿瘤的发展提供支持^[79]^。此外，肿瘤与神经元之间的相互作用，不仅影响肿瘤的生长模式，也在一定程上改变了神经元的行为，形成一个恶性循环（图1）。

**图1:**
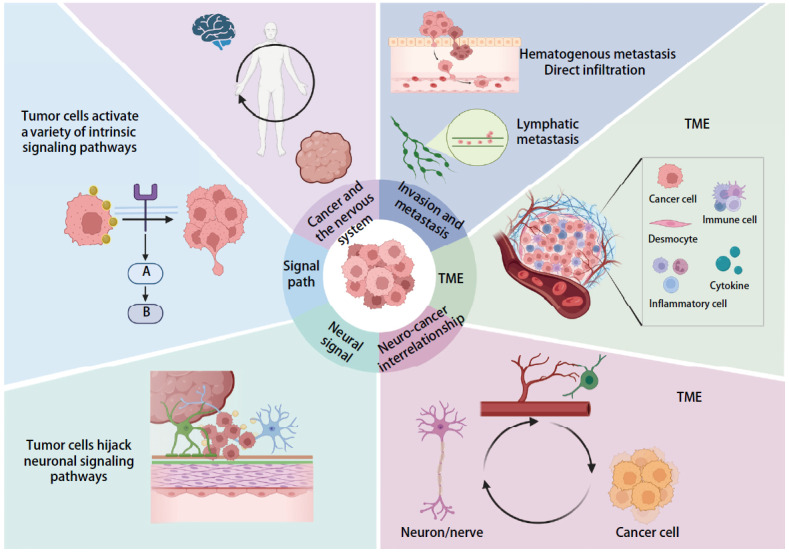
肿瘤细胞与神经信号在肿瘤转移中的串扰作用。（1）肿瘤细胞信号激活：模式图左侧展示了肿瘤细胞如何激活多种内在信号通路；（2）TME：右侧部分详细描绘了TME，包括肿瘤细胞、免疫细胞、成纤维细胞和炎症细胞，以及它们之间的相互作用；（3）肿瘤细胞的转移途径：模式图右上角展示了肿瘤细胞的主要转移方式：血行转移、淋巴道转移以及直接浸润；（4）肿瘤与神经系统的相互作用：模式图底部左侧和右侧分别展示了肿瘤细胞如何劫持神经元信号传导途径，以及神经元与肿瘤细胞之间的相互作用；（5）TME与神经系统的串扰：模式图右下部分强调了TME与神经系统之间的复杂相互作用，这种串扰可能影响肿瘤的侵袭和转移行为。本模式图综合展示了肿瘤细胞、TME和神经系统之间的复杂相互作用，这些相互作用对肿瘤的发展和转移具有重要影响（使用BioRender.com网站在线绘制）。

## 4 小结与展望

随着技术的进步，我们可以期待更深入的研究来探索肿瘤与神经系统的相互作用。对NEC的谱系可塑性机制的研究，可为其精准治疗提供潜在的治疗靶标，对NEC的谱系可塑性机制在NEC谱系可塑性的过程中，基因突变、转录因子和表观遗传修饰互相调节，构成了错综复杂的调节网络，需要更多的研究来验证潜在的药物靶点和临床疗效。例如，开发针对通讯劫持机制的靶向治疗，可能为脑转移患者带来新的治疗选择。未来的研究需要更加系统和综合的方法来整合不同的观点和发现，以期绘制出更为清晰的癌症神经科学全景图。

总之，深入了解肿瘤细胞的通信劫持作用，以及与神经信号的串扰作用，随着癌症神经科学研究的不断发展，未来的研究将为癌症的预防和治疗提供新的希望，我们将在这方面进行前沿的探索。
